# Pornografie im Alltag und in der sexuellen Bildung von Jugendlichen: Befragungsergebnisse aus 8. und 9. Schulklassen in Nordrhein-Westfalen

**DOI:** 10.1007/s00103-025-04073-x

**Published:** 2025-07-09

**Authors:** Nicola Döring, Veronika Mikhailova, Matthias Biermann, Yasemin Bresemann, Alexander Daum, Tanita Kistinger, Mareike Wellner, Thomas Zenge Wesendonk

**Affiliations:** 1 Fakultät für Wirtschaftswissenschaften und Medien, Technische Universität Ilmenau, Ehrenbergstraße 29, 98693 Ilmenau, Thüringen Deutschland; 2 AWO Beratungszentrum für Familienplanung, Schwangerschaftskonflikte und Fragen der Sexualität, Essen, Nordrhein-Westfalen Deutschland; 3 AWO Fachstelle Jugendarbeit und Sexualpädagogik Münster, Münster, Nordrhein-Westfalen Deutschland; 4 Beratungsstelle für sexuelle Bildung und HIV/STI-Prävention im AWO-Kreisverband Wuppertal e. V., Wuppertal, Nordrhein-Westfalen Deutschland; 5 AWO Beratungsstelle für Schwangerschaftskonflikte, Familienplanung, Paar- und Lebensberatung Dortmund, Dortmund, Nordrhein-Westfalen Deutschland; 6 AWO Beratungsstelle für Schwangerschaft und Schwangerschaftskonflikte, Familienplanung, Sexualität und Partnerschaft im Kreis Wesel, Dinslaken, Nordrhein-Westfalen Deutschland

**Keywords:** Pornografie, Sexualaufklärung, Sexualpädagogik, Sexuell explizite Online-Inhalte, Sexuelle Sozialisation, Pornography, Sexuality education, Sex education, Sexually explicit online content, Sexual socialization

## Abstract

**Hintergrund:**

Im Digitalzeitalter nehmen Kontakte Minderjähriger mit Online-Pornografie zu. Aktuelle Daten für Deutschland fehlen jedoch.

**Ziel der Arbeit:**

Daher war es Ziel der vorliegenden Studie, die Verbreitung ungewollter und gewollter Pornografiekontakte unter Jugendlichen (Forschungsfrage 1, F1), ihre Einstellungen zu Pornografie (F2), ihre Vorstellungen von gutem Sex (F3) sowie ihren subjektiven Bedarf an Aufklärung über Pornografie (F4) zu erfassen.

**Material und Methoden:**

Zwischen 2023 und 2024 wurden Jugendliche der 8. und 9. Jahrgangsstufe an verschiedenen weiterführenden Schulen Nordrhein-Westfalens im Klassenverband schriftlich befragt. Von *N* = 903 Jugendlichen (85 % 14–15 Jahre; 54 % männlich, 85 % heterosexuell) liegen vollständige Datensätze vor.

**Ergebnisse:**

Sowohl ungewollte (48 %) als auch gewollte (63 %) Pornografiekontakte waren bei den befragten Minderjährigen weitverbreitet (F1). Ihre Einstellungen zu Pornografie waren zwiespältig: Sie schätzten sie beispielsweise als erregend und schädlich, als süchtig und Spaß machend ein (F2). In ihren Vorstellungen von gutem Sex spielten pornountypische Aktivitäten (z. B. Küssen, Streicheln am ganzen Körper, Liebeserklärungen) eine deutlich größere Rolle als pornotypische (z. B. hartes Stoßen, Würgen am Hals, Analverkehr, F3). Die befragten Minderjährigen äußerten Bedarf an Aufklärung über Pornografie, etwa dazu, wie sie wirkt (58 %) und wie realistisch sie ist (55 %, F4). Unterschiede zwischen Jungen und Mädchen sowie Vielnutzenden und Wenignutzenden werden aufgezeigt.

**Diskussion:**

Um Jugendliche mit ihren sexuell expliziten Medienerfahrungen nicht allein zu lassen, ist es sinnvoll, Pornografie als Thema in der sexuellen Bildung alters- und sachgerecht aufzugreifen.

## Abstract (en)

**Background:**

In the digital age, minors’ exposure to online pornography is increasing. However, current data for Germany are lacking.

**Research aim:**

The aim of this study was to examine the prevalence of unwanted and wanted pornography exposure among adolescents (research question 1, RQ1), their attitudes toward pornography (RQ2), their conceptions of what constitutes good sex (RQ3), and their subjective need for education about pornography (RQ4).

**Material and methods:**

Between 2023 and 2024, students in the 8th and 9th grades from various secondary schools in North Rhine-Westphalia were surveyed in a classroom setting using questionnaires. Complete datasets are available for *N* = 903 adolescents (85% aged 14–15 years; 54% male, 85% heterosexual).

**Results:**

Both unwanted (48%) and wanted (63%) exposure to pornography were widespread among the surveyed minors (RQ1). Their attitudes toward pornography were ambivalent: they described it as arousing yet harmful, as addictive yet fun (RQ2). In their conceptions of good sex, activities atypical of pornography (e.g., kissing, full-body caressing, declarations of love) played a greater role than those typical of pornography (e.g., hard thrusting, choking, anal intercourse, RQ3). The surveyed minors expressed a need for education about pornography, including its effects (58%) and its realism (55%, RQ4). Differences between boys and girls and between heavy and light pornography users are highlighted.

**Discussion:**

To ensure adolescents are not left alone with their experiences of sexually explicit media, it is advisable to address pornography as a topic in sexual education in an age-appropriate and factual manner.

## Hintergrund

*Pornografie* ist ein Sammelbegriff für Mediendarstellungen, die 3 Kriterien erfüllen [1–4]: (1) Sie stellen Sexualität mit Fokus auf Sexualpraktiken und Genitalien sehr detailliert dar (Inhaltskriterium); (2) sie zielen darauf ab, das Publikum sexuell zu erregen (Funktionskriterium); (3) ihre Produktion und Verbreitung erfolgt mit informierter Einwilligung der involvierten erwachsenen Darstellenden (Konsenskriterium). Das Konsenskriterium verweist darauf, dass Pornografie Ergebnis legaler Sexarbeit (professionelle Pornografie) oder freiwilliger Freizeitaktivitäten (Amateurpornografie) unter Erwachsenen ist [5, 6]. So prüft die aktuell populärste Pornografieplattform PornHub (betrieben von Aylo; früher: MindGeek), dass alle bereitgestellten Videos Erwachsene zeigen, die informierte Einwilligung zur Erstellung und Verbreitung des Materials gegeben haben; PornHub distanziert sich damit von früheren Geschäftspraktiken, die das Konsenskriterium nicht ausreichend sichergestellt haben [7]. Die Forschung plädiert für eine klare Abgrenzung zwischen einvernehmlich produzierten und verbreiteten sexuellen Mediendarstellungen einerseits („Pornografie“) und andererseits solchen Darstellungen, die nicht konsensuell erzeugt und verbreitet werden und daher Straftatbestände und Gewalt darstellen („Missbrauchsabbildungen“, „bildbasierte sexuelle Gewalt“ [8]).

Im Digitalzeitalter ist Pornografie in großer Menge und Vielfalt jederzeit und überall zugänglich, und zwar nicht nur für erwachsene, sondern auch für minderjährige Mediennutzende. So finden sich Pornografieplattformen wie PornHub unter den 30 meistbesuchten Websites weltweit [9] und können meist problemlos von Minderjährigen abgerufen werden. Auch über soziale Medien, wie beispielsweise X (früher: Twitter) mit einem Mindestalter von 13 Jahren in den USA und 16 Jahren in Deutschland, wird sexuell expliziter Content verbreitet. Der rechtliche und technische Kinder- und Jugendmedienschutz greift im globalen Internet nur sehr begrenzt. Eltern haben zwar die Möglichkeit, den Zugang zu Pornografie über das häusliche WLAN und die Endgeräte ihrer Kinder zu beschränken (z. B. DNS-(Domain-Name-System‑)Filter, Betriebssystemeinstellungen, Kinderschutz-Apps), inwiefern sie diese Möglichkeiten ausschöpfen und Kinder diese Maßnahmen umgehen (z. B. durch Nutzung der Endgeräte von Freund*innen), ist jedoch unbekannt.

Die Tatsache, dass Minderjährige heutzutage im Internet sehr leicht mit pornografischen Darstellungen in Kontakt kommen können, hat zu kontroversen öffentlichen und fachlichen Debatten geführt [10]. Teilweise ist von einer „Pornoseuche“ die Rede [11]. Die heutigen Jugendlichen werden zuweilen als „Generation Porno“ [12] bezeichnet, da sie sexuell explizite Mediendarstellungen konsumieren, lange bevor sie erste eigene sexuelle Erfahrungen sammeln. Dies wird theoretisch und empirisch mit Gefahren wie sexueller Verrohung, Verunsicherung und sexuellen Fehlvorstellungen in Zusammenhang gebracht sowie auch mit Sucht, negativem Körperbild, Leistungsdruck und einer Verfestigung geschlechtlicher Stereotypisierung, Hierarchisierung und Gewalt (z. B. [13–16]). Unterschiedliche Medienwirkungstheorien wie etwa die sozialkognitive Theorie, die sexuelle Skripttheorie oder der Kultivierungsansatz gehen von Lerneffekten auf mehreren Ebenen aus, vor allem davon, dass pornografische Vorbilder sexuelles Wissen, sexuelle Einstellungen und sexuelle Verhaltensweisen beeinflussen [17].

Während die einen von starken negativen Wirkungen der Pornografie auf Jugendliche ausgehen, warnen andere vor einer neuen Moralpanik (z. B. [18]). Sie argumentieren theoretisch und empirisch, dass Jugendliche Pornografie sehr unterschiedlich nutzen, Inhalte bewusst auswählen oder meiden und durchaus kritisch einordnen können, indem sie zwischen fiktiver Pornowirklichkeit und realer Partnersexualität unterscheiden (z. B. [19]), schließlich wirken neben der Pornografie auch weiterhin viele andere Faktoren auf das jugendliche Sexualitätsverständnis (z. B. familiäre Sozialisation, Peer-Erfahrungen, schulische und außerschulische sexuelle Bildungsangebote, kulturelle und religiöse Werte). Zudem lassen sich pornografische Inhalte als fiktionale Darstellungen ebenso wie sexuelle Fantasien nicht nur als handlungsleitende Wunschvorstellungen, sondern teilweise auch als kompensatorische Fantasien verstehen, die erregen, obwohl (oder weil) ihre reale Umsetzung ausdrücklich nicht gewünscht ist [10, 20].

*Pornografiekompetenz* als Fähigkeit, sexuell explizites Medienmaterial entsprechend den eigenen Bedürfnissen bewusst zu meiden oder auszuwählen und kritisch einzuordnen [21], wird nicht nur als präventiv für negative Pornografiewirkungen betrachtet. Diskutiert und empirisch aufgezeigt werden in der Fachliteratur auch positive Effekte der Beschäftigung mit Pornografie (z. B. [22–25]): Dazu zählen Lustgewinn und die Befriedigung sexueller Neugier, Selbstakzeptanz, wenn Pornografie verdeutlicht, dass man mit den eigenen Fantasien und Vorlieben nicht allein dasteht, Abbau von Unsicherheiten, Schuldgefühlen und Ängsten sowie Zugewinn an Handlungskompetenz, wenn man schon einmal gesehen hat, wie bestimmte sexuelle Techniken funktionieren.

So kontrovers die Wirkungsdebatten rund um Jugendliche und Pornografie geführt werden, so schmal ist die zugrunde liegende Datenbasis. Nur wenige Längsschnittstudien und so gut wie keine Experimentalstudien mit Minderjährigen liegen vor, die Kausalaussagen erlauben würden [26]. Verfügbare Studien sind in der Regel rein korrelativer Natur und selbst diese sind rar, wenn man auf konkrete kulturelle Kontexte und Altersgruppen fokussiert. So existiert unseres Wissens keine aktuelle, qualitätsgeprüfte wissenschaftliche Studie für Deutschland zum alltäglichen Umgang Jugendlicher mit Pornografie und zu ihren Anforderungen an Pornografieaufklärung. Diese Forschungslücke will der vorliegende Beitrag schließen. Er fasst zunächst den aktuellen Forschungsstand mit Fokus auf Deutschland zusammen und berichtet dann Ergebnisse einer aktuellen Befragung unter gut 900 Jugendlichen zu ihren Kontakten mit Pornografie, ihren Einstellungen zu Pornografie, ihren Vorstellungen von gutem Sex und ihren Anforderungen an Pornografieaufklärung. Damit aktualisiert der Beitrag den bisherigen Forschungsstand und gibt der Praxis der sexuellen Bildung Impulse dazu, ob und wie Pornografie im Unterricht thematisiert werden sollte.

## Forschungsstand

Mit der Veralltäglichung des Internets ist das internationale Forschungsinteresse an jugendlichen Pornografiekontakten in den letzten Dekaden stark gewachsen. Inzwischen liegen einige systematische Übersichtsarbeiten vor, die den bisherigen Forschungsstand zusammenfassen (z. B. [4, 27, 28]). Der aktuellste systematische Forschungsüberblick zum Einfluss von Pornografie auf Kinder und Jugendliche schließt *k* = 166 zwischen den Jahren 2000 und 2022 veröffentlichte Studien ein [29]. Er berichtet jedoch entgegen dem Versprechen im Titel des Artikels keine Daten zur Kausalität, sondern lediglich Korrelationen und betont, dass selbst diese oft widersprüchlich sind, etwa wenn intensivere Pornografienutzung in manchen Studien mit schlechteren, in anderen Studien dagegen mit besseren Schulnoten korreliert.

Die genannten Forschungsübersichten [4, 27–29] kommen zu dem Schluss, dass es große Unterschiede im Umgang mit Pornografie zwischen Jugendlichen gibt, die u. a. mit dem Geschlecht, der sexuellen Identität, dem Alter und dem kulturellen Kontext in der Weise zusammenhängen, dass männliche, nichtheterosexuelle und ältere Jugendliche aus westlichen Ländern mehr Pornografienutzung berichten. Weiterhin weisen die genannten Forschungsübersichten darauf hin, dass sich Angebot und Nutzung von Pornografie durch den digitalen Medienwandel stark verändert haben in dem Sinn, dass Angebot und Nutzung tendenziell zunehmen.

Wir konnten insgesamt 15 Studien aus den letzten 20 Jahren finden, die Daten zur Pornografienutzung von Jugendlichen in Deutschland liefern.^1^ Diese lassen sich anhand ihrer Methodik bezüglich Datenerhebung und Stichprobenbildung in 3 Gruppen einteilen:

### Mündliche Befragungen (4 Studien).

Dazu gehören eine qualitative Studie mit *N* = 35 Jugendlichen (13–19 Jahre), die in Fokusgruppen über Pornografie diskutierten [30], eine qualitative Interviewstudie mit *N* = 160 Jugendlichen (16–19 Jahre) aus Hamburg und Leipzig [31, 32] sowie eine standardisierte, bundesweit repräsentative Interviewstudie unter *N* = 1229 Kindern und Jugendlichen (11–17 Jahre), die von einem Umfrageinstitut im Auftrag der Jugendzeitschrift „Bravo“ aus dem Bauer-Verlag durchgeführt wurde [33]. Die Daten dieser 3 mündlichen Befragungsstudien sind jeweils rund 15 Jahre alt. Die repräsentative BZgA(Bundeszentrale für gesundheitliche Aufklärung)/BIÖG(Bundesinstitut für Öffentliche Gesundheit)-Trendstudie zur Jugendsexualität, die mündliche und schriftliche Befragungsteile umfasst, erfragte zuletzt vor rund 5 Jahren in ihrer 9. Welle, ob Jugendliche aus Pornografie („Sexfilmen“) etwas für sie Wichtiges über Sexualität erfahren haben [34].

### Online-Befragungen (5 Studien).

3 Studien waren auffindbar, bei denen im Internet Selbstselektionsstichproben von Jugendlichen rekrutiert und per Online-Fragebogen untersucht wurden: Dabei konnten im Jahr 2007/2008 *N* = 352 Jugendliche (16–19 Jahre) erreicht werden [35], im Jahr 2011 *N* = 1077 Kinder und Jugendliche (12–21 Jahre; [36]) und im Jahr 2014 *N* = 302 Jugendliche (14–17 Jahre; [37]). Diese Daten sind 10 bis 17 Jahre alt. Zudem war eine über 5 Jahre alte Befragung identifizierbar, die mit einer Quotenstichprobe aus einem Online-Panel arbeitete und im Jahr 2017 *N* = 1048 Jugendliche und junge Erwachsene (14–20 Jahre) einschließen konnte [38]. Eine parallel zu unserer Studie durchgeführte Befragung im Auftrag der Landesanstalt für Medien Nordrhein-Westfalen (NRW) erfragte 2023 und 2024 (jeweils *N* ≈ 3000) die Pornografieerfahrungen von Kindern (11–13 Jahre) und Jugendlichen (14–17 Jahre) aus einem Online-Panel, wobei keine qualitätsgeprüfte wissenschaftliche Veröffentlichung in einem Fachjournal vorliegt, sondern nur ein selbstveröffentlichter Bericht [39].

### Schriftliche Befragungen im Klassenverband (6 Studien).

Weiterhin waren 6 Umfragen identifizierbar, bei denen der Forschungsfragebogen jeweils (auch) in Schulklassen verteilt wurde: Das Spektrum reicht von Studien mit Teilnehmendenzahlen im dreistelligen [40–42] bis unteren vierstelligen Bereich [43]. An der Studie „EU Kids Online“ beteiligten sich in der zweiten Welle (2017–2019) 19 Länder mit Erhebungen in Schulen und Privathaushalten, wobei für Deutschland Daten von 12- bis 16-Jährigen aus Privathaushalten vorliegen [44]. Die sächsische Trendstudie PARTNER erhob zuletzt vor rund 5 Jahren in ihrer 5. Welle Daten zur Pornografienutzung unter *N* = 861 16- bis 18-Jährigen [45].

Typische Themen der genannten 15 Jugend-Pornografie-Studien aus Deutschland, von denen nur 4 in begutachteten Fachzeitschriften publiziert wurden [31, 32, 35, 40], sind die Verbreitung von ungewollten und gewollten Pornografiekontakten unter Jugendlichen, ihre Einstellungen und Fragen zu sowie Reaktionen auf Pornografie. In Übereinstimmung mit dem internationalen Forschungsstand [4, 27–29] weisen auch die Daten aus Deutschland auf große Unterschiede im Umgang mit Pornografie zwischen Jugendlichen hin, etwa in Abhängigkeit von Geschlecht und Alter. Auf einzelne Befunde der genannten früheren Studien werden wir später zurückkommen, um unsere aktuellen Daten zu kontextualisieren.

## Forschungsziel

Ziel der vorliegenden Studie war es, den Status quo des Umgangs mit Pornografie unter Jugendlichen in Deutschland zu erfassen, um Forschungslücken zu schließen und die Praxis der sexuellen Bildung zu informieren. Dabei beziehen sich die ersten beiden Forschungsfragen (F1 und F2) auf Verbreitung und Einstellungen:

- F1: Wie verbreitet sind ungewollte und gewollte Pornografiekontakte unter Jugendlichen?
- F2: Welche Einstellungen haben Jugendliche zur Pornografie?

Darüber hinaus sollten mit Blick auf mögliche Inhalte der sexuellen Bildung potenziell durch Pornografie beeinflusste Vorstellungen von gutem Sex (F3) sowie Anforderungen an die Pornografieaufklärung erfasst werden (F4):

- F3: Welche sexuellen Praktiken und Merkmale gehören nach Auffassung der Jugendlichen zu gutem Sex?
- F4: Welche Anforderungen haben Jugendliche an Pornografieaufklärung?

## Methode

Die Darstellung der Methode umfasst a) Untersuchungsdesign und Forschungsethik, b) Stichprobe, c) Instrument sowie d) Datenerhebung und Datenanalyse.

### Untersuchungsdesign und Forschungsethik

Bei der vorliegenden Studie handelt es sich um eine standardisierte Fragebogenerhebung im Klassenverband, wobei der Fragebogen per Smartphone oder auf Papier ausgefüllt werden konnte. Die Teilnahme an der von der Ethikkommission der Technischen Universität Ilmenau genehmigten Studie erfolgte vollkommen freiwillig und basierte auf informierter Einwilligung der Jugendlichen (ab 14 Jahre), ihrer Eltern und der Schulleitungen. Fragebogen, Datensatz und statistisches Analyseskript sind öffentlich verfügbar (siehe Datenverfügbarkeitserklärung).

### Stichprobe

Die Rekrutierung der Stichprobe erfolgte hauptsächlich (85 %) in der 8. und 9. Jahrgangsstufe unterschiedlicher weiterführender Schulen in NRW im Rahmen des sexualpädagogischen Projektunterrichts, der von externen sexualpädagogischen Fachkräften unterschiedlicher Träger durchgeführt wird. Die vorliegende Studie arbeitete mit Fachkräften der Sexualpädagogik der Arbeiterwohlfahrt (AWO) und mit Youthwork NRW (https://youthwork-nrw.de/). Von *N* = 903 Jugendlichen (85 % 14–15 Jahre, 54 % männlich, 85 % heterosexuell) liegen vollständige Datensätze vor (Tab. 1).

**Tab. 1:** Soziodemografische Angaben zur Stichprobe (*N* = 903) als absolute Häufigkeiten und Prozentwerte

Variable	*n*	%
*Klassenstufe*		
8. Klasse	187	21
9. Klasse	583	64
Andere Klassenstufe	133	15
*Schulform*		
Hauptschule	34	4
Realschule	59	7
Sekundarschule	5	< 1
Gesamtschule	206	23
Gymnasium	599	66
*Alter*		
14	392	43
15	376	42
16	124	14
17	11	1
*Geschlecht*		
Weiblich	400	44
Männlich	487	54
Nichtbinär	8	< 1
Divers	4	< 1
Anderes	4	< 1
*Sexuelle Orientierung*		
Heterosexuell	772	85
Homosexuell	18	2
Bisexuell	36	4
Pansexuell	17	2
Asexuell	12	1
Anderes	5	< 1
Weiß noch nicht	43	5

Prozentwerte sind gerundet. Von den 903 vollständigen Fällen gingen 752 über den Online-Fragebogen und 151 über den Papierfragebogen ein

Gut die Hälfte der Befragten (52 %) gab an, dass beide Eltern aus Deutschland stammen, und 73 %, an einen Gott zu glauben. Bisherige romantische und sexuelle Erfahrungen verteilten sich in der Stichprobe alterstypisch [46]: 68 % hatten sich schon einmal selbst befriedigt, 44 % hatten schon einmal eine feste Beziehung und 14 % hatten schon einmal Sex.

### Instrument

Das Fragebogeninstrument wurde deduktiv auf der Basis des bisherigen Forschungsstandes und induktiv auf der Basis der Praxiserfahrung der involvierten sexualpädagogischen Fachkräfte entwickelt und besteht aus 56 Items in 5 Fragenblöcken: a) Angaben zur Person, b) ungewollte und gewollte Pornografiekontakte (F1), c) Einstellungen zur Pornografie (F2), d) Vorstellungen von gutem Sex (F3) und e) Anforderungen an Pornografieaufklärung (F4). Der Fragebogen gab folgende Gegenstandsdefinition vor: „Im Internet gibt es verschiedene Arten von Pornografie. Unter Pornografie (= Pornos) versteht man Videos, Fotos und Geschichten von Menschen, die Sex haben. Jetzt geht es darum, was du davon bislang mitbekommen hast.“

Der Fragebogen wurde einer Augenscheinvalidierung durch sexualpädagogische Fachkräfte und einem empirischen Pretest unterzogen. Er sollte für Schüler*innen aller weiterführenden Schulformen (ohne Förderschulen) leicht und schnell (binnen ca. 10 min) zu beantworten sein. Daher musste auf psychometrische Skalen verzichtet und auf eine Erhebung mit Einzelitems zurückgegriffen werden. Der Online-Fragebogen wurde mit der Umfragesoftware *ESF Survey* programmiert für ein Ausfüllen per Smartphone. Für Schüler*innen, die in der Befragungssituation kein Smartphone verfügbar hatten, wurde alternativ eine Papierversion des Fragebogens bereitgestellt. Schüler*innen, die nicht an der Studie teilnehmen wollten, konnten sich den Fragebogen online oder auf Papier ohne Option zum Ausfüllen anschauen.

### Datenerhebung und Datenanalyse

Die Datenerhebung erfolgte zwischen Februar 2023 und August 2024 im Rahmen des sexualpädagogischen Projektunterrichts durch externe sexualpädagogische Fachkräfte. Ohne vorherige Behandlung des Pornografiethemas erfolgte die Bearbeitung am Ende einer Doppelstunde vor der Pause. Damit wurde den Schüler*innen in der Pause ermöglicht, die Befragung zu reflektieren und mit Peers zu besprechen. Nach der Pause bestand dann die Möglichkeit, Fragen zu stellen und das Thema Pornografie sexualpädagogisch aufzugreifen. Alle Fachkräfte, die die Befragung im Unterricht durchführten, taten dies in standardisierter Form gemäß einer Checkliste nach einer Online-Schulung.

Die Datenanalyse erfolgte deskriptivstatistisch über Prozent- und Mittelwerte sowie inferenzstatistisch über zweidimensionale Chi-Quadrat-Tests und eindimensionale Varianzanalysen mit dem Statistikpaket R. Angesichts der großen Stichprobe wurde ein Signifikanzniveau von 1 % zugrunde gelegt.

## Ergebnisse

Die Ergebnisse werden gegliedert nach den 4 Forschungsfragen berichtet.

### Ungewollte und gewollte Pornografiekontakte

Knapp die Hälfte der befragten Jugendlichen (48 %) berichtete, im Internet schon einmal *ungewollt* auf Pornografie gestoßen zu sein, und rund ein Drittel (34 %) gab an, schon einmal von einer anderen Person ungewollt Pornografie zugeschickt oder gezeigt bekommen zu haben. Hinsichtlich der ungewollten Kontakte traten bei der Lebenszeitprävalenz keine statistisch signifikanten Geschlechterdifferenzen auf.

Die Lebenszeitprävalenz für *gewollte* Pornografiekontakte lag bei 63 % mit signifikanter Geschlechterdifferenz: 83 % der befragten Jungen und 69 % der geschlechterdiversen Jugendlichen hatten schon absichtlich im Internet Pornografie gesucht und gesehen gegenüber 38 % der Mädchen (*χ*^*2*^(2) = 184,53, *p* = < 0,001, *V* = 0,45).

**Abb. 1:**
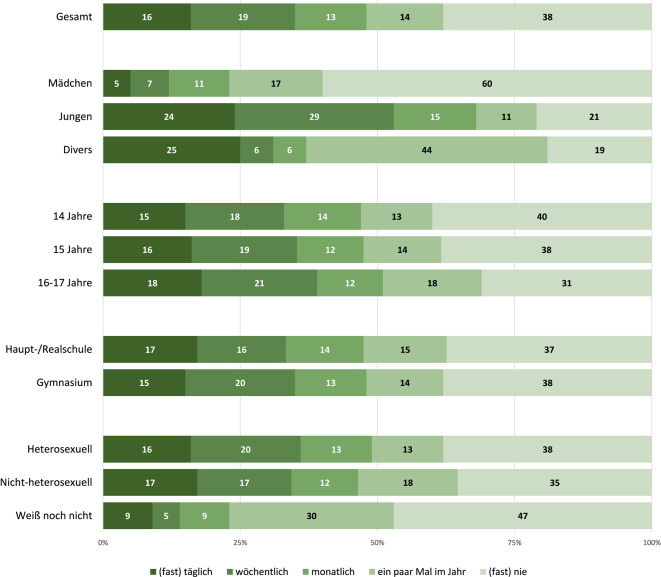
Häufigkeit der Pornografienutzung unter den befragten Jugendlichen (*N* = 903) in den letzten 12 Monaten als Prozentwerte der Häufigkeitskategorien. (Quelle: eigene Abbildung)

Signifikante Differenzen gemäß Geschlecht zeigten sich auch bei der *Häufigkeit* der gewollten Pornografienutzung in den letzten 12 Monaten (Abb. 1). So berichteten 24 % der Jungen und 25 % der diversen Jugendlichen gegenüber 5 % der Mädchen tägliche Nutzung (*F*(1; 901) = 223,25, *p* < 0,001, *η*^*2*^_*G*_ = 0,20). Die Differenzen bei Alter, Schultyp und sexueller Identität waren nicht statistisch signifikant.

Viele Jugendliche suchten absichtlich nach Pornografie im Internet und berichteten teilweise auch, dass sie schon Pornografieinhalte gefunden hatten, die ihnen *gefielen*, wobei sich signifikante Geschlechtsunterschiede zeigten: So gaben 75 % der Jungen, 62 % der diversen Jugendlichen und 24 % der Mädchen an, schon Pornovideos gesehen zu haben, die ihnen gefielen (*χ*^*2*^(2) = 226,61, *p* < 0,001, *V* = 0,50). Gleichzeitig hatten 66 % der Jungen, 50 % der diversen Jugendlichen und 16 % der Mädchen schon Pornofotos gesehen, die ihnen gefielen (*χ*^*2*^(2) = 222,87, *p* < 0,001, *V* = 0,50). Pornografische Geschichten, die ihnen gefielen, kannten 62 % der diversen Jugendlichen und 39 % der Mädchen gegenüber 20 % der Jungen (*χ*^*2*^(2) = 49,40, *p* < 0,001, *V* = 0,23).

### Einstellungen zur Pornografie

Die Jugendlichen äußerten zwiespältige Einstellungen zur Pornografie: Sie hielten sie beispielsweise für erregend und schädlich, für süchtig und Spaß machend (Tab. 2).

**Tab. 2:** Einstellungen zur Pornografie unter den befragten Jugendlichen (*N* = 903) nach Geschlecht und Häufigkeit der Pornografienutzung als Prozentwerte

Pornos …	Gesamt (*N* = 903) %	Geschlecht^a^			Häufigkeit der Pornografienutzung		
		Jungen (*n* = 487) %	Mädchen (*n* = 400) %	*V*^b^	Vielnutzende (*n* = 432) %	Wenignutzende (*n* = 471) %	*V*^b^
… sind erregend	62	82	38	0,45***	91	35	0,57***
… sind für Jugendliche schädlich	58	52	66	0,13***	46	70	0,24***
… machen süchtig	55	61	47	0,14***	65	45	0,19***
… machen Spaß	54	66	38	0,28***	80	28	0,52***
… sind eine Sünde	48	49	47	0,02	43	52	0,09**
… sind generell schädlich	47	44	51	0,07	38	55	0,18***
… sind eklig	46	32	62	0,30***	21	69	0,47***
… stumpfen sexuell ab	45	47	43	0,04	50	41	0,09**
… sind gewalttätig	39	30	50	0,20***	32	45	0,13***
… sind frauenfeindlich	37	27	49	0,23***	29	44	0,15***
… zeigen wie guter Sex abläuft	19	23	16	0,09**	25	14	0,13***
… sind realistisch	15	17	12	0,06	15	15	0,00
… sind männerfeindlich	13	11	15	0,05	10	16	0,09**

Vielnutzende = *tägliche/wöchentliche/monatliche Nutzung; Wenignutzende* *=* *ein paar Mal im Jahr/(fast) nie*

Ganzzahlig gerundete Prozentwerte für „Ja“-/„Eher-Ja“-Antworten

Die Tabelle ist absteigend nach den Prozentwerten in der Gesamtspalte sortiert

^a^Geschlechterdiverse Jugendliche wurden wegen der geringen Fallzahl (*n* = 16) nicht in diese Analyse einbezogen

^b^Ergebnisse des zweidimensionalen Chi-Quadrat-Tests: Cramers V‑Werte als Effektgrößenmaß, ***p < 0,001, **p < 0,01, das Signifikanzniveau lag bei 1 %

Dabei zeigten sich statistisch signifikante Unterschiede zwischen Jungen und Mädchen sowie zwischen Viel- und Wenignutzenden in der Richtung, dass Jungen und Vielnutzende den Lust- und Spaßfaktor deutlich stärker betonten (große Effektgrößen), Mädchen und Wenignutzende dagegen den Ekelfaktor (mittlere bis große Effektgrößen). Zudem betonten Mädchen stärker den Aspekt der Gewalt und Frauenfeindlichkeit (mittlere Effektgrößen). Wenignutzende und Mädchen schrieben der Pornografie mehr Schädlichkeit zu (kleiner Effekt), Vielnutzende und Jungen betonten stärker den Suchtfaktor (kleiner Effekt). Dass Pornos realistisch sind (15 %) oder zeigen, wie guter Sex abläuft (19 %), glaubten nennenswerte Teilgruppen der Befragten (Tab. 2).

### Vorstellungen von gutem Sex

Von den befragten, überwiegend sexuell unerfahrenen Jugendlichen (siehe oben Beschreibung der Stichprobe) nannten mehr als 3 Viertel Küssen, Vaginalverkehr, Orgasmus des Mannes und der Frau sowie Streicheln am ganzen Körper als Elemente von gutem Sex (Tab. 3). Zwischen Jungen und Mädchen sowie zwischen Viel- und Wenignutzenden von Pornografie bestand insofern Einigkeit, dass für die meisten von ihnen Küssen und Liebeserklärungen zu gutem Sex gehören.

**Tab. 3:** Vorstellungen von gutem Sex unter den befragten Jugendlichen (*N* = 903) nach Geschlecht und Häufigkeit der Pornografienutzung als Prozentwerte

Zu gutem Sex gehört …	Gesamt (*N* = 903) %	Geschlecht^a^			Häufigkeit der Pornografienutzung		
		Jungen (*n* = 487) %	Mädchen (*n* = 400) %	*V*^b^	Vielnutzende (*n* = 432) %	Wenignutzende (*n* = 471) %	*V*^b^
… Küssen	95	94	97	0,07	97	94	0,07
… Vaginalverkehr (Geschlechtsverkehr)	90	92	88	0,07	94	86	0,14***
… Orgasmus des Mannes	84	87	81	0,07	90	78	0,16***
… Orgasmus der Frau	84	86	82	0,05	89	79	0,14***
… Streicheln am ganzen Körper	83	79	88	0,11***	85	81	0,05
… Streicheln an den Geschlechtsorganen (Petting)	75	77	72	0,05	84	66	0,20***
… Liebeserklärungen	63	62	65	0,03	61	65	0,03
… viele verschiedene Stellungen	58	66	50	0,16***	69	48	0,22***
… Dirty Talk (sexuelle Ausdrücke sagen)	52	55	49	0,06	63	43	0,20***
… langes Durchhalten des Mannes	46	58	33	0,24***	54	39	0,16***
… Oralverkehr beim Mann (Blasen)	44	59	27	0,31***	61	29	0,32***
… lautes Stöhnen der Frau	43	56	29	0,26***	56	32	0,24***
… Oralverkehr bei der Frau (Lecken)	42	49	35	0,14***	55	30	0,25***
… dominantes Verhalten des Mannes	42	43	40	0,02	49	35	0,14***
… hartes Stoßen	35	40	30	0,10**	44	27	0,18***
… großer Penis des Mannes	32	40	23	0,17***	41	23	0,19***
… Schläge auf den Po	29	37	19	0,20***	38	20	0,20***
… Würgen am Hals	24	21	27	0,07	27	20	0,08
… Analverkehr (Po-Sex)	23	34	10	0,28***	31	17	0,16***
… große Brüste der Frau	19	42	9	0,38***	38	16	0,25***

Vielnutzende = *tägliche/wöchentliche/monatliche Nutzung; Wenignutzende* *=* *ein paar Mal im Jahr/(fast) nie*

Ganzzahlig gerundete Prozentwerte für „Ja“-/„Eher-Ja“-Antworten

Die Tabelle ist absteigend nach den Prozentwerten in der Gesamtspalte sortiert

^a^Geschlechterdiverse Jugendliche wurden wegen der geringen Fallzahl (*n* = 16) nicht in diese Analyse einbezogen

^b^Ergebnisse des zweidimensionalen Chi-Quadrat-Tests: Cramers V‑Werte als Effektgrößenmaß, ***p < 0,001, **p < 0,01, das Signifikanzniveau lag bei 1 %

Darüber hinaus zeigten sich kleine bis mittlere statistisch signifikante Unterschiede in den Vorstellungen von gutem Sex zwischen Jungen und Mädchen sowie zwischen Viel- und Wenignutzenden von Pornografie. Die größten Geschlechterdifferenzen bestanden dahin gehend, dass Jungen stärker als Mädchen große Brüste der Frau (*V* = 0,38***), Oralverkehr beim Mann (*V* = 0,31***), Analverkehr (*V* = 0,28***) und lautes Stöhnen der Frau (*V* = 0,26***) als Bestandteile von gutem Sex ansahen (Tab. 3). Jungen und Mädchen sowie Viel- und Wenignutzende von Pornografie unterschieden sich im Gesamtmuster dahin gehend, dass Jungen und Vielnutzende stärkere Zustimmung zu allen zur Auswahl gestellten Merkmalen sexueller Interaktionen zeigten.

### Anforderungen an Pornografieaufklärung

Die befragten Jugendlichen zeigten unabhängig vom Geschlecht relativ großes Interesse daran, Pornografieaufklärung zu erhalten: Mehr als die Hälfte würde gern mehr darüber erfahren, wie Pornos auf Jugendliche wirken, wie realistisch sie sind, welchen Einfluss sie auf die eigene Sexualität haben können sowie was erlaubt und verboten ist im Hinblick auf Pornografie (Tab. 4).

**Tab. 4:** Anforderungen an Pornografieaufklärung unter den befragten Jugendlichen (*N* = 903) nach Geschlecht und Häufigkeit der Pornografienutzung als Prozentwerte

Ich würde gern mehr darüber erfahren …	Gesamt (*N* = 903) %	Geschlecht^a^			Häufigkeit der Pornografienutzung		
		Jungen (*n* = 487) %	Mädchen (*n* = 400) %	*V*^b^	Vielnutzende (*n* = 432) %	Wenignutzende (*n* = 471) %	*V*^*b*^
… wie Pornos auf Jugendliche wirken	58	57	60	0,03	63	53	0,10**
… was realistisch und was unrealistisch ist an Pornos	55	51	60	0,09**	61	50	0,11***
… welchen Einfluss Pornos auf meine Sexualität haben können	53	56	49	0,06	62	45	0,17***
… was erlaubt und verboten ist im Hinblick auf Pornos	51	48	55	0,07	58	45	0,13***
… unter welchen Bedingungen Pornos produziert werden	45	43	48	0,04	53	38	0,14***
… wie Jugendliche Pornos nutzen	41	43	37	0,06	49	33	0,16***

Vielnutzende = *tägliche/wöchentliche/monatliche Nutzung; Wenignutzende* *=* *ein paar Mal im Jahr/(fast) nie*

Ganzzahlig gerundete Prozentwerte für „Ja“-Antworten

Die Tabelle ist absteigend nach den Prozentwerten in der Gesamtspalte sortiert

^a^Geschlechterdiverse Jugendliche wurden wegen der geringen Fallzahl (*n* = 16) nicht in diese Analyse einbezogen

^b^Ergebnisse des zweidimensionalen Chi-Quadrat-Tests: Cramers V‑Werte als Effektgrößenmaß, ***p < 0,001, **p < 0,01, das Signifikanzniveau lag bei 1 %

Vielnutzende äußerten durchgängig statistisch signifikant mehr Interesse an Pornografieaufklärung als Wenignutzende (Tab. 4).

## Diskussion

Die Diskussion gliedert sich in die Ergebnisinterpretation, Limitationen der Studie und Fazit.

### Interpretation der Befunde

Ungewollte und gewollte Pornografiekontakte sind laut der vorliegenden Studie weitverbreitet, wobei die intentionale Nutzung überwiegt und bei einer nennenswerten Gruppe von Jugendlichen (nämlich bei 24 % der Jungen und 5 % der Mädchen) täglich stattfindet (F1, Abb. 1). Dagegen hatte eine im Jahr 2014, also 10 Jahre früher, durchgeführte Befragung derselben Altersgruppe 9 % tägliche Nutzung bei Jungen und 1 % bei Mädchen festgestellt [37]. Im Einklang mit dem internationalen Forschungsstand [29] zeigt sich also auch für Deutschland sowohl eine Zunahme der gewollten Pornografiekontakte als auch eine Tendenz zum Schließen des Gender-Gaps in dem Sinn, dass die Pornografienutzung der Mädchen stärker steigt und sich somit der der Jungen langsam annähert. Gefallen fanden die befragten Mädchen und geschlechterdiversen Jugendlichen insbesondere an Textpornografie, die seit dem Erfolg der Romantrilogie „Fifty Shades of Grey“ im Jahr 2011 kulturell deutlich sichtbarer geworden ist, sowohl als kommerzielle Literatur (Bücher der Genres *Erotic Romance* und *Dark Romance*) als auch als selbstproduzierte *Erotic Fan Fiction* [47].

Jugendliche äußerten differenzierte Einstellungen zur Pornografie (F2). Sie sehen das Spaß- und Lustpotenzial, benennen aber auch Ekel, Gefahren wie Sucht und religiös-moralische Ablehnung. Schließlich werden Pornografienutzung und Selbstbefriedigung bei strenger Auslegung der religiösen Regeln von Christentum, Judentum und Islam größtenteils abgelehnt. So stimmten 48 % der Befragten der Aussage „Pornos sind eine Sünde“ zu, 37 % beurteilten Pornos als frauenfeindlich und 13 % als männerfeindlich (Tab. 2). In einer Vorläuferstudie mit Schüler*innen in NRW mit einer etwas breiteren Altersspanne [42] waren die moralischen Bedenken wegen Sünde (12 %), Frauenfeindlichkeit (26 %) und Männerfeindlichkeit (7 %) geringer verbreitet. In Übereinstimmung mit früheren Studien [37] hielt eine nennenswerte Teilgruppe der befragten Jugendlichen Pornografie für realistisch (15 %) und vorbildlich für guten Sex (19 %).

Das Bild, das die überwiegend sexuell unerfahrenen befragten Jugendlichen von gutem Sex hatten, lässt sich insgesamt nicht als pornografietypisch beschreiben (Tab. 3, F3). Insbesondere alarmistische Behauptungen, dass Jugendliche sich im eigenen Sexualverhalten so stark an Pornografie orientieren, dass sie sich schon gar nicht mehr küssen [11], steht im Widerspruch zu unserem Befund, dass Küssen unabhängig von Geschlecht und Pornografienutzung bei allen Befragten das meistgenannte Element (95 %) von gutem Sex war und sich auch bei Liebeserklärungen (63 %) keine signifikanten Gruppenunterschiede zeigten. Insbesondere Jungen und Vielnutzende betrachteten ein breiteres Spektrum an Merkmalen und Aktivitäten als Bestandteile von gutem Sex, darunter auch Elemente pornografischer Skripte (z. B. langes Durchhalten des Mannes, lautes Stöhnen der Frau, Oralverkehr beim Mann, große Brüste der Frau, großer Penis des Mannes, Analverkehr), einschließlich härterer Sexualpraktiken [48], wie z. B. dominantes Verhalten des Mannes, hartes Stoßen, Schläge auf den Po und Würgen am Hals (Tab. 3). Über Kausalrichtungen können unsere Daten jedoch keine Auskunft geben. Theoriebasiert ist es plausibel, von wechselseitigen Beeinflussungen auszugehen: Wer breitere und intensivere sexuelle Interessen hat, wird sich oft stärker der Pornografie zuwenden, und wer viel Pornografie nutzt, kann Interessen an einem breiteren Spektrum an sexuellen Aktivitäten entwickeln [37].

Jugendliche – und insbesondere Vielnutzende – äußerten mehrheitlich Interesse an einer Behandlung des Themas Pornografie in der sexuellen Bildung (Tab. 4, F4). Dieser Befund stützt erneut die Empfehlungen aus der Fachliteratur, die Pornografiekompetenz von Jugendlichen in Angeboten der sexuellen Bildung zu fördern [21, 49].

### Limitationen

Die Stärke der vorliegenden Studie besteht darin, dass sie aktuelle Daten einer relativ großen Stichprobe liefert und die Pornografienutzung im Alltag mit Anforderungen an die Pornografieaufklärung verbindet. Die Befragung im Klassenverband in NRW ist allerdings nicht repräsentativ für Jugendliche in NRW oder Deutschland. Förderschulen wurden nicht eingeschlossen. Gymnasien sind überrepräsentiert, sodass die Befunde eher Erfahrungen von Jugendlichen mit höherer Schulbildung darstellen. Geschlechterdiverse Jugendliche sind in zu kleiner Fallzahl im Sample enthalten, um aussagekräftige Analysen zu dieser Gruppe zu erlauben. Das Fragebogeninstrument bestand aus Einzelitems, deren psychometrische Eigenschaften unbekannt sind. Da im Sinne der Forschungsethik nur Jugendliche eingeschlossen werden konnten, bei denen Schulleitungen, Eltern und die Jugendlichen selbst eine informierte Einwilligung zur Teilnahme an der Pornografiestudie gaben, ist mit Verzerrungen zu rechnen, die jedoch in Summe nicht genau abschätzbar sind. Anzunehmen ist beispielsweise, dass Eltern und Schulleitungen, die Pornografienutzung bei ihren Jugendlichen a) für nicht bzw. kaum existent oder aber b) für ein gravierendes Problem halten, die Teilnahme eher abgelehnt haben, da sie die Datenerhebung womöglich für a) überflüssig oder b) kompromittierend halten.

### Fazit

Für Institutionen und Fachkräfte der sexuellen Bildung ergibt sich angesichts der in der vorliegenden Studie aufgezeigten hohen Prävalenzen und Häufigkeiten von Pornografiekontakten die Herausforderung, die Erfahrungen Jugendlicher mit sexuell expliziten Medieninhalten nicht zu ignorieren, sondern alters- und sachgerecht aufzugreifen. Das ist umso wichtiger, als Lehrkräfte und Eltern sich eine umfassende sexuelle Bildung für Jugendliche wünschen, selbst aber oft davor zurückscheuen, Themen wie Pornografie, Selbstbefriedigung und Sexualpraktiken zu besprechen. Im Rahmen sexueller Bildung wären beispielsweise unterschiedliche Vorstellungen von Bestandteilen von gutem Sex zu thematisieren (z. B. Oral- und Analverkehr, Schläge und Würgen), einschließlich der dabei in der Praxis notwendigen Konsensaushandlungen und Sicherheitsvorkehrungen, die in pornografischen Darstellungen der entsprechenden Praktiken meist ausgeblendet bleiben [10].

Die Behandlung des Pornografiethemas durch ausgebildete sexualpädagogische Fachkräfte gewinnt zudem vor dem Hintergrund des dynamischen Medienwandels weiter an Relevanz. So wirft nach der Etablierung der Online-Pornografie nun auch das Aufkommen der synthetischen (d. h. durch Tools der generativen künstlichen Intelligenz erzeugten) Pornografie neue Probleme auf, wie etwa die Verbreitung nichtkonsensueller Deepfake-Pornografie, bei der das Gesicht von unbeteiligten Personen (z. B. Mitschüler*innen, Lehrkräften, Prominenten) ohne deren Zustimmung täuschend echt in pornografisches Material integriert wird [50].

Die Finanzierung umfassender sexueller Bildung ist in Deutschland politisch und rechtlich bislang nicht flächendeckend gesichert. Es geht also darum, professionelle sexuelle Bildung im Präsenzformat der schulischen Sexualkunde zu sichern und auszubauen. Um den zunehmenden sexuellen Mediengebrauch, einschließlich gewollter und ungewollter Pornografiekontakte, angemessen zu behandeln mit dem Ziel, einen verantwortungsvollen und konsensuellen Umgang mit Pornografie und Sexualität zu fördern, braucht es genügend Zeit und Raum. Weiterhin kann es sinnvoll sein, qualitätsgesicherte Online-Ressourcen zur Pornografieaufklärung zu entwickeln, auf die Jugendliche bei Bedarf niedrigschwellig und diskret zugreifen können [10].

## Data Availability

Die Studie folgt dem Open-Science-Ansatz, das heißt, der Fragebogen, der anonyme Datensatz und das statistische Analyseskript sind vollständig öffentlich verfügbar (https://osf.io/mnxpy/).

## Footnotes

1 Die regelmäßig wiederholten bevölkerungsrepräsentativen Jugend- und Medien-Studien in Deutschland behandeln Pornografienutzung nicht (z. B. Shell-Jugendstudie, mpfs-JIM-Studie, ARD/ZDF Onlinestudie).
